# C-reactive protein to lymphocyte ratio as a new biomarker in predicting surgical site infection after posterior lumbar interbody fusion and instrumentation

**DOI:** 10.3389/fsurg.2022.910222

**Published:** 2022-10-04

**Authors:** Xiaofei Wu, Xun Ma, Jian Zhu, Chen Chen

**Affiliations:** ^1^ Department of Orthopaedic Surgery, Third Hospital of Shanxi Medical University, Shanxi Bethune Hospital, Shanxi Academy of Medical Sciences, Tongji Shanxi Hospital, Taiyuan, China; ^2^ Department of Orthopaedic Surgery, The Third Hospital of Hebei Medical University, Shijiazhuang, China

**Keywords:** posterior lumbar interbody fusion, perioperative management, risk prediction tool, creative protein to lymphocyte count ratio (CLR), surgical site infection

## Abstract

**Purpose:**

This study aims to evaluate the potential of C-reactive protein to lymphocyte count ratio (CLR) for the prediction of surgical site infection (SSI) following posterior lumbar interbody fusion (PLIF) and the instrumentation of lumbar degenerative diseases.

**Methods:**

In this retrospective study, we considered patients with a lumbar degenerative disease diagnosis surgically treated by the instrumented PLIF procedure from 2015 to 2021. Patient data, including postoperative early SSI and other perioperative variables, were collected from their respective hospitalization electronic medical records. The receiver operator characteristic curve was constructed to determine the optimal cut-off value for CLR, and the ability to predict SSI was evaluated by the area under the curve (AUC). According to the cut-off value, patients were dichotomized with high- or low-CLR, and between-group differences were compared using univariate analysis. The independent impact of CLR on predicting SSI was investigated by multivariate logistics regression analysis.

**Results:**

A total of 773 patients were included, with 26 (3.4%) developing an early SSI post-operation. The preoperative CLR was 11.1 ± 26.1 (interquartile range, 0.4–7.5), and the optimal cut-off was 2.1, corresponding to a sensitivity of 0.856, a specificity of 0.643, and an AUC of 0.768 (95% CI, 0.737–0.797). CLR demonstrated a significantly improved prediction ability than did lymphocyte count (*P *= 0.021) and a similar ability to predict an infection as C-response protein (*P* = 0.444). Patients with a high CLR had a significantly higher SSI incidence than those with a low CLR (7.6% vs. 0.8%, *P *< 0.001). After adjustment for numerous confounding factors, CLR ≥ 2.1 was associated with an 11.16-fold increased risk of SSI, along with other significant variables, i.e., diabetes, preoperative waiting time, and surgical duration.

**Conclusion:**

A high CLR exhibited an improved ability to predict incident SSI and was associated with a substantially increased risk of SSI following instrumented PLIF. After better-design studies verified this finding, CLR could potentially be a beneficial tool in surgical management.

## Introduction

Postoperative surgical site infection (SSI) remains a major issue after spinal surgeries, despite adequate prophylactic antibiotics being routinely administered before and after surgery (1). Compared to other approaches, the instrumented posterior lumbar interbody fusion (PLIF) procedure is more likely to be affected by postoperative SSI, and the incidence rate was reported to vary from 1.5% to 7.2% (2–6). SSI is an intractable issue that is resistant to antibiotics in half of the cases, whereby 30% necessitated revision surgery or implant removal (3). Furthermore, even if managed promptly and appropriately, patients with SSI would have greater long-term back pain and less than half of the probability (27% vs. 60%) of achieving a minimum clinically important difference compared to those without (7). Besides, the substantial economic burden from prolonged hospitalization stays, readmission for revision procedures, and nursing care significantly impacted patients and their families (8, 9).

The preoperative identification of patient and clinical factors or biomarkers that effectively predict the postoperative SSI can inform risk evaluation and stratification, facilitating the implementation of targeted prevention measures, which should thus aid in the avoidance of excessive medical resource consumption and the chance of resultant antibiotic resistance. In contrast with patient and clinical factors that are sometimes subjective and obtuse in showing body status (e.g., body's response to tissue injury, inflammatory, and immune status), and SSI, serum biomarkers are more sensitive, objective, and prompt (10). For example, C-response protein (CRP) is a biomarker and is not only a typical acute phase reactant protein in response to inflammation but also an indicator of injury duration in the face of repeated tissue injury (11). The ability of elevated serum CRP concentration to predict SSI after spinal surgeries has been extensively demonstrated in recent studies (12, 13), which was appraised as “more predictive than prehistoric” (13). However, false negatives were often encountered for various reasons, including the low sensitivity in low-virulence-bacterial infections where serum CRP concentration was low (14, 15). Similar observations have also been shown for another biomarker, lymphocyte count (12, 16, 17), which is also particularly important for the immune response state. However, a previous study determined the optimal cut-off of both CRP and lymphocyte to be within the normal reference range, e.g., 4.4 mg/L (reference range, <8 mg/L) for CRP, and 1.2* *×* *10^9^/L (reference range, 1.1–3.2* *×* *10^9^/L) for lymphocyte count, respectively (12), thus limiting their clinical viability. In other words, the “seemingly normal” value for a biomarker is underpowered to alert the treating surgeons to the increased risk of SSI following surgery.

We conducted this study by considering their indicative value of inflammatory/immune status, the demonstrated ability to predict SSI, and their inherent limitations. We hypothesize that CRP to lymphocyte ratio (CLR), derived from both biomarkers, is a better index than predicting SSI after instrumented PLIF. We also hypothesize that high CLR is independently associated with an increased risk of SSI.

## Methods

This retrospective study was performed following the Helsinki Declaration. The study protocol was approved by the ethics committee of the local institution, which waived the need for informed consent because of the identification anonymity.

Patient electronic medical records were retrieved to identify those who underwent an instrumented PLIF procedure for a lumbar degenerative disease, i.e., degenerative disc disease, spondylolisthesis, spinal stenosis, or a combination of the above, in our hospital, between January 2015 and December 2021. The inclusion criteria were age ≥18 years and complete medical records. The exclusion criteria were procedures other than an instrumented PLIF, obvious symptoms, signs, or preexisting conditions directly affecting the preoperative CRP concentration or lymphocyte count (e.g., respiratory or urinary tract infection, autoimmune hepatitis, liver cirrhosis, rheumatoid arthritis, tumor, etc.), past surgery at lumbar vertebra, primary or metastatic lumbar tumor, or incomplete medical records.

The instrumented PLIF procedure was performed with total facetectomy and subtotal intervertebral discectomy for adequate posterior decompression, cages with local or allergenic bone graft inserted into the intervertebral space, and fixation of a fused segment with a screw-rod system. The operations were performed by six orthopedic or spinal surgeons. As per the standard guidelines, prophylactic intravenous single-dose cephalosporins (e.g., cefazolin and cefamandole nafate) were routinely administered 30 min prior to skin incision. For operative procedures exceeding 3 h, an additional dose would be given. After the operation, prophylactic antibiotics were routinely administered. However, the duration relied on the perceived individualized risk of infection, often one to three days and occasionally up to one week, which was at the discretion of their treating surgeons.

### Identification of SSI cases

Reviewing the electronic medical records, we identified early SSIs during hospitalization. The US Center for Disease Control and Prevention 2017 was used to diagnose and classify SSI (18). A superficial SSI refers to an infection involving skin and subcutaneous tissues with possible symptoms or signs (i.e., redness, tenderness, heat, and pain over the wound site) and can be resolved by local wound care and antibiotics treatment without the need for surgical intervention. Deep SSI refers to an infection involving the deep issue (i.e., fascia, muscle tissues, or vertebra space), with resultant marked serious symptoms/signs (e.g., fever, pain, tenderness, persistent wound discharge or dehiscence, abscess or gangrenosis), often requiring surgical intervention.

### Calculation of CLR and measurements

Blood sampling and testing were performed following the manufacturer's instructions. CLR was calculated by dividing the serum CRP concentration in mg/L by the lymphocyte count in 10^9^/L. A preoperative blood sample was extracted to obtain the measurements. For patients with multiple measurements for biomarkers of interest (including CRP, lymphocyte count, and the below-mentioned ones), the one closest to the operation was chosen to minimize the time-dependent effect. Using the manufacturer's recommended cut-offs, these biomarkers were interpreted, and the normal range was <8 mg/L or 1.10–3.20* *×* *10^9^/L for CRP and lymphocytes.

### Variables of interest

Two researchers (XW and XM) extracted the variables of interest from the medical records. These included socioeconomic features (age, gender, type of insurance), lifestyle (current smoking, alcohol drinking), comorbidities [body mass index (BMI) calculated by dividing body weight in kilograms by square of height in meters, diabetes, hypertension, heart disease, cerebrovascular disease, chronic obstructive pulmonary disease (COPD), renal insufficiency, peripheral vascular disease, past any operation in the lumbar spine], surgery-related variables [preoperative waiting time, American Society of Anesthesiologists (ASA) score, operated levels, surgical duration, intraoperative bleeding, allogeneic blood transfusion, allograft bone use, postoperative prophylactic use of antibiotics], and laboratory test results [albumin, white blood cell (WBC), neutrophil, lymphocyte, red blood cell (RBC) and platelet count, hematocrit, hemoglobin, and fasting blood glucose (FBG)].

### Statistical analysis

Continuous data were presented with a mean ± standard deviation (SD), and their normality status was detected employing a Kolmogorov–Smirnov test. A Student *t*-test or Mann–Whitney-*U* test was performed based on the normality status, as appropriate. Categorical data were presented with figures and percentage values, and a between-group comparison was performed by a Chi-square test or Fisher's exact test.

The optimal cut-off value of CLR to predict SSI was determined by the receiver operating characteristic (ROC) curve when the Youden index (specificity plus sensitivity −1) was maximized. The corresponding sensitivity, specificity, and area under the ROC curve (AUC) with a 95% confidence interval (95% CI) were calculated. Additionally, a similar method was used for CRP and lymphocyte count for comparison purposes. The AUCs for three biomarkers were pairwise compared by a Z-test (19), using the MedCalc software version 14.8.1 (MedCalc Software Ltd, Ostend, Belgium).

Based on the above-determined optimal cut-off value of CLR, patients were dichotomized into the high- or low-CLR groups, and the differences were detected by univariate analysis. Variables tested with *P* < 0.10 during univariate analysis were further selected for adjustment in the multivariate logistic regression model, using the “enter” method to minimize the confounding effects. The magnitude of CLR associated with SSI was indicated by odds ratios (ORs) and 95% CI. The goodness-of-fit of the multivariate model was evaluated by the Hosmer–Lemeshow (H–L) test, with *P *> 0.05 indicating an acceptable result and a higher Nagelkerke R^2^ value (normal range, <1.0) suggesting a better result. *P *< 0.05 was set as the statistical significance level. The analysis was performed using SPSS 26.0 (IBM, Armonk, New York, USA).

## Results

There were 773 patients (348 males and 425 females), with an average age of 51.8 ± 12.8 years. The mean preoperative stay was 3.3 ± 2.4 days, and the operating level was 2.1 ± 1.8. Of the patients, 13.8% (107/773) received an allogeneic bone or bone substitute graft, and 32.7% (253/773) received an intraoperative allogeneic transfusion. The surgical time for the procedure was 175.6 ± 51.1 min, and approximately half (45.8%, 354/773) had a procedure lasting above 3 h. Postoperatively, prophylactic antibiotics use ≥3 days was administered in 21.1% (163/773) of the patients. In total, 26 (3.4%) patients had an early SSI postoperatively, including 12 (1.6%) deep and 14 (1.8%) superficial SSIs.

**Figure 1:**
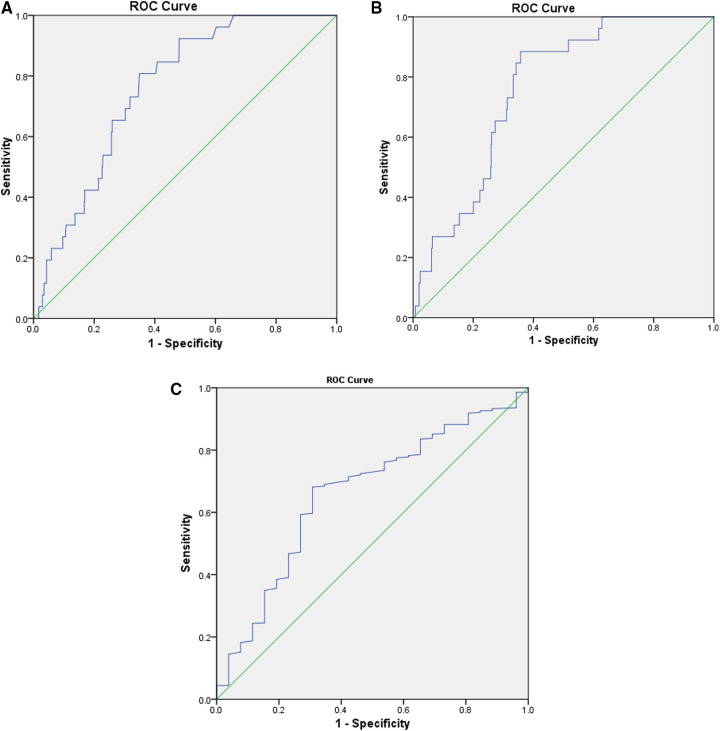
The ROC curves were constructed to determine the optimal cut-off values for CLR, CRP, and lymphocyte count. The optimal cut-off of CLR was 2.1, corresponding to a sensitivity of 0.856, specificity of 0.643, and an AUC of 0.768 (95% CI, 0.737–0.797) (**A**). The optimal value of CRP was 4.0 mg/L, corresponding to a sensitivity of 0.808, specificity of 0.651, and AUC of 0.759 (95% CI, 0.727–0.789) (**B**); while for lymphocyte count, the cut-off value was 1.5, and the sensitivity, specificity, and AUC was 0.681, 0.692, and 0.660 (95% CI, 0.555–0.765) (**C**). AUC, area under the curve; CLR, C-reactive protein to lymphocyte ratio; CRP, C-response protein; ROC, receiver operator characteristic.

The preoperative CLR was 11.1 ± 26.1, with a range of 0–215.8 (interquartile range, 0.4–7.5). The ROC curve determined the optimal cut-off as 2.1; the corresponding sensitivity and specificity were 0.856 and 0.643, respectively; the AUC was 0.768 (95%CI, 0.737–0.797). Patients with a high CLR had a significantly higher SSI incidence rate than those with a low CLR (7.6%, 22/289 vs. 0.8%, 4/484; crude OR = 9.2; *P *< 0.001). The optimal value of CRP was 4.0 mg/L, corresponding to the sensitivity, specificity, and AUC of 0.808, 0.651, and 0.759 (95% CI, 0.727–0.789), respectively. Meanwhile, the cut-off value for lymphocyte count was 1.5, and the sensitivity, specificity, and AUC were 0.681, 0.692, and 0.660 (95% CI, 0.555–0.765), respectively (Figure 1). The Z-test demonstrated a significantly improved prediction ability of CLR compared to that of lymphocyte count (Z value, 2.309; *P* = 0.021), but was nonsignificant compared to CRP (Z value, 0.765; *P* = 0.444). It was nonsignificantly different from CRP with lymphocyte count (Z value, 1.723; *P* = 0.085).

Patients with a high CLR value were significantly different from those with a low CLR value in terms of age in the form of either continuous (*P *= 0.007) or categorical variables (*P *= 0.006), prevalence of obesity (*P *= 0.031), hypertension (*P *= 0.031), diabetes (*P *= 0.036), peripheral vascular disease (*P *< 0.001), preoperative waiting time (*P *< 0.001), allograft bone (*P *< 0.001), intraoperative bleeding (*P *< 0.001), allogenic blood transfusion (*P *< 0.001), surgical duration (*P *= 0.010), WBC count (*P* < 0.001), albumin <35 g/L (*P *< 0.001), FBG > 6.1 mmol/L (*P *< 0.001), neutrophil count >6.3* *×* *10^9^/L (*P *< 0.001), lymphocyte count (*P *< 0.001), RBC count (*P *< 0.001), hemoglobin (*P *< 0.001), and hematocrit (*P *< 0.001) (Table 1).

**Table 1:** Univariate analysis of factors associated with CLR.

Variables	Number (%) of patients with CLR ≥ 2.1 (*n* = 289)	Number (%) of patients with CLR < 2.1 (*n* = 484)	*P*
Gender (male)	132 (45.7)	216 (44.6)	0.777
Age (year)	53.3 ± 13.7	50.8 ± 12.2	0.007
<45	67 (23.2)	136 (28.1)	0.006
45-64	158 (54.7)	283 (58.5)	
≥65	64 (22.1)	65 (13.4)	
BMI	25.9 ± 4.0	25.5 ± 3.4	0.095
Obesity (BMI ≥ 28 kg/m^2^)	78 (27.0)	98 (20.2)	0.031
Hypertension	93 (32.2)	122 (25.2)	0.036
Diabetes mellitus	50 (17.3)	48 (9.9)	0.003
Heart disease	22 (7.6)	28 (5.8)	0.318
COPD	14 (4.8)	21 (4.3)	0.744
Cerebrovascular disease	23 (8.0)	37 (7.6)	0.875
Peripheral vascular disease	40 (13.8)	22 (4.5)	<0.001
Preoperative waiting time	4.5 ± 3.1	2.7 ± 1.6	<0.001
Total hospital stay	16.1 ± 6.1	12.7 ± 3.9	<0.001
Current smoking	61 (21.1)	93 (19.2)	0.524
Alcohol drinking	87 (30.1)	150 (31.0)	0.796
Previous operation in any site	58 (20.1)	123 (25.4)	0.090
Operated levels	2.2 ± 1.7	2.1 ± 1.9	0.715
allogeneic bone or bone substitute			<0.001
No	210 (72.7)	456 (94.2)	
Yes	79 (27.3)	28 (5.8)	
ASA score			0.076
I	19 (6.6)	50 (10.3)	
II–IV	270 (93.4)	434 (89.7)	
Intraoperative bleeding (ml)	771.2 ± 390.5	546.8 ± 292.8	<0.001
Allogenic blood transfusion	132 (45.7)	121 (25.0)	<0.001
Surgical duration (minutes)	171.8 ± 55.4	162.0 ± 48.1	0.010
Postoperative antibiotic use ≥3 days	57 (19.7)	106 (21.9)	0.473
WBC (>10* *×* *10^9^/L)	146 (50.5)	40 (8.3)	<0.001
Albumin (<35 g/L)	100 (34.6)	5 (1.0)	<0.001
FBG (>6.1 mmol/L)	130 (45.0)	76 (15.7)	<0.001
Neutrophil (>6.3* *×* *10^9^/L)	184 (63.7)	49 (10.1)	<0.001
Lymphocyte (<1.1* *×* *10^9^/L)	109 (37.7)	23 (4.8)	<0.001
Platelet (>300* *×* *10^9^/L)	29 (10.0)	41 (8.5)	0.464
RBC (<Lower limit)	86 (29.8)	16 (3.3)	<0.001
Hemoglobin (<Lower limit)	75 (26.0)	27 (5.6)	<0.001
Hematocrit (<Lower limit)	143 (49.5)	50 (10.3)	<0.001

**Obesity**, defined as a BMI ≥ 28 kg/m^2^, in accordance with the criteria fitted for Chinese adults. RBC reference range: female, 3.5–5.0 × 10^12^/L; males, 4.0–5.5 × 10^12^/L; hemoglobin reference range: females, 110–150 g/L; males, 120–160 g/L; hematocrit reference range: females, 35%–45%; males, 40%–50%.

COPD, chronic obstructive pulmonary disease; CLR, C-response protein to lymphocyte ratio; BMI, body mass index; ASA, American Society of Anesthesiologists; FBG, fasting blood glucose; WBC, white blood cell; RBC, red blood cell.

The multivariate logistic regression analysis, adjusted for the above significant variables and those with *P *< 0.10 (BMI in continuous form, ASA score, and history of any operation), displayed that CLR ≥ 2.1 was associated with an 11.16-fold increased risk of SSI. The other significant variables included diabetes (OR, 3.31; 95% CI, 1.04–9.55), preoperative waiting time in a day (OR, 1.16; 95% CI, 1.01–1.35), and surgical duration in each 30-min increment (OR, 1.24; 95% CI, 1.06–1.59) (Table 2). The H–L test showed an acceptable goodness-of-fit of the multivariate model (*P* = 0.537, Chi-square = 6.993; Nagelkerke R^2 ^= 0.273).

**Table 2:** Multivariate analysis of CLR in association with SSI after adjustment for numerous variables^a^.

Variables	OR (95% CI)	*P*
CLR ≥ 2.1	11.16 (3.71–27.43)	<0.001
Diabetes	3.31 (1.04–9.55)	0.014
Preoperative waiting time (each day increment)	1.16 (1.01–1.35)	0.048
Surgical duration (each 30-min increment)	1.24 (1.06–1.59)	0.029

^a^ Multivariate model adjusted for age, hypertension, diabetes, peripheral vascular disease, history of any surgery, preoperative waiting time, allogeneic bone graft, ASA score, allogenic blood transfusion, surgical duration, BMI, albumin, FBG, WBC, neutrophils, RBC, HGB, and HCT.

CLR, C-response protein to lymphocyte ratio; SSI, surgical site infection; OR, odds ratio; CI, confidence interval; BMI, body mass index; ASA, American Society of Anesthesiologists; FBG, fasting blood glucose; WBC, white blood cell; RBC, red blood cell; HGB, hemoglobin; HCT, hematocrit.

## Discussion

We verified our previous study reported that preoperative high CLR value (≥2.1) was significantly associated with an 11.16-fold risk of SSI following instrumented PLIF for lumbar degenerative disease. We also found that CLR indicated a better predicting ability, with an AUC of 0.768, a significant difference for lymphocyte count (AUC, 0.660; *P* = 0.021), but nonsignificant for CRP (AUC, 0.759; *P* = 0.444). CLR revealed a higher sensitivity than the original index (CLR, 0.856; CRP, 0.808; and lymphocyte count, 0.681).

SSI is a disastrous complication after spinal orthopedics or other surgeries, and exploring the potential new indexes has been a primary task in clinical research. However, existing risk prediction models based on identified clinical risk factors demonstrated less robustness in predicting postoperative SSI (2, 20–22). The underlying reasons are related to the heterogeneous population and the time-dependent confounding effects of biomarkers. On the other hand, patient self-reported comorbidities as a component of a risk prediction model were a contributor since these self-reported medical conditions may not mirror the true pathophysiological basis. An ideal prediction tool should be readily accessible, easy to use, and rely upon preoperatively routinely measured laboratory parameters. Inflammation/immune biomarkers fit these characteristics well, and more importantly, they are often highly sensitive to the body's pathophysiologic response and have been presenting notable changes before clinical signs or manifestations emerge (23).

During the past decade, numerous derived novel biomarkers have been employed in research and in clinical practice, demonstrating good prognostication for clinical outcomes or complications, including Modified Glasgow Prognostic Score (mGPS), neutrophil to lymphocyte ratio (NLR), platelet to lymphocyte ratio (PLR), CRP to albumin ratio (CAR), systemic immune-inflammation index (SII), fibrinogen to albumin ratio (FAR), lymphocyte to monocyte ratio (LMR), and monocyte to high-density lipoprotein ratio, among others (24–28). As for CLR or lymphocyte to CRP ratio (LCR), the previous studies on surgical tumors (osteosarcoma, gastric cancer, lung cancer, or pancreatic cancer) (29–31) and on infectious events following surgeries (32–34) have demonstrated its effectiveness in providing prognostic information. To the best of our knowledge, this study was the first to apply CLR in spinal orthopedic surgeries to predict the incidence of postoperative SSI.

In this study, CLR demonstrated better predictive ability than the original index, lymphocyte count, or CRP, with AUC increasing from 0.660 and 0.808 to 0.868 (*P* value, 0.021 and 0.444). This suggests that the predictive effect of this new biomarker was remarkably strengthened after the division calculation, which was related to the simultaneous uptrend of CRP and downtrend of the lymphocyte count. Most importantly, the predictive effect *via* this cut-off (≥2.1) is, albeit related to CRP and lymphocyte count, incompletely dependent on either one taken individually. In other words, CLR could still exceed the cut-off value even if both biomarkers are simultaneously in reference intervals. The identified optimal cut-off value of CRP and lymphocyte count was exactly within the range of the manufacturer's reference interval (CRP: cut-off, 4.0 mg/L; reference interval, <8 mg/L; lymphocyte count, cut-off, 1.5* *×* *10^9^/L; reference interval, 1.1 to 3.2* *×* *10^9^/L). In clinical practice, applying this seemingly normal value as a cut-off for either CRP or lymphocyte count is hardly possible to alert healthcare providers of the substantially increased risk of postoperative SSI. Therefore, CLR can be considered a pragmatic and independent predictive tool.

The other clinical importance of using CLR is guiding postoperative administration. In this study, CLR ≥ 2.1 corresponds to a sensitivity of 0.856, suggesting that patients with a CLR < 2.1 are at low risk (0.8%, 4/484) of postoperative SSI and can thus be considered to execute “no antibiotic strategy” or “less use strategy” postoperatively, to reduce the possibility of multiple drug-resistant bacteria. It is worth noting that CLR's specificity is only 0.643, suggesting a high probability of false positive results. Therefore, a positive CLR result is a determiner of active preventive interventions; combined systemic medical conditions and local operative conditions (i.e., lumbar disease *per se*) should be evaluated for an informed decision.

The results show that preoperative CLR, derived from CRP and lymphocyte count, is a feasible and predictive biomarker for the early incidence of SSI following instrumented PLIF procedures for degenerative lumbar diseases. An elevated CLR ≥ 2.1 was independently associated with an 11.15-fold risk of SSI. This value may alert surgeons of the high risk of postoperative SSI, better facilitating the implementation of feasible targeted preventive measures.

## Data Availability

The data analyzed in this study is subject to the following licenses/restrictions: In accordance with the institutional policy, data used in this study are not available publicly but can be obtained from the corresponding author upon justified request for scientific research purposes. Requests to access these datasets should be directed to Xun Ma, xunmadoc1776@126.com.
